# Notes and News

**Published:** 1887-12-03

**Authors:** 


### Notes and News.

Collected and Communicated.

Dr. Moody Ward has been appointed physician to the Royal Berkshire Hospital.

We stated last week that the Matron of the Liverpool Eye and Ear Infirmary was asking for contributions of under-clothing for children. We are requested to state that the institution requiring warm underclothing is the St. Paul's Eye and Ear Infirmary, Liverpool.

The following are the *ipsissima verba* of the author of a scheme in aid of the London Hospitals. He writes: "I have a scheme which can be tested on a small scale first and which will realise a prophet *(sic)* on every 50s. invested a nett profit of £1 . 13 . 4 . No risks or bad debts. That would be = on every £100 = to £46 . 13 . 4 . nett. I shall be glad to give you further particulars by interview. I have worked the matter out carefully and would give my time voluntary and can get others to assist."

We regret to hear that, in consequence of somewhat failing strength, Mr. Septimus Lowe, J.P., intends resigning his office as one of the surgeons of the Lincoln County Hospital. Mr. Lowe has rendered great services to the hospital for many years, and it is expected that he will be appointed consulting surgeon to the institution.

Lovers of dogs, and those who care for their well-being, will be pleased to read the following: "The Australian Mail just to hand gives particulars of the awards at the Adelaide International Exhibition, by which we see that Spratt's Patent, Limited, as usual, has obtained the premier position, receiving the highest award. The Saltaire Exhibition, just closed, has also awarded this company a gold medal for their unique exhibits of dog, poultry, and game houses and appliances."

The introductory address in connection with the winter session at the Meath Hospital was delivered by Dr. A. Wynne Foot in the lecture hall of the institution. There was a large gathering of medical men and students. Sir George Owens, M.D., presided, and among those present were Sir Charles Cameron, M.D., Sir William Stokes, Dr. Little (president of the College of Physicians), Dr. A. H. Coley (president of the College of Surgeons), and many other distinguished members of the medical profession.

Of modern inventions not the least curious is the baby-hatching machine, which has lately been introduced into some Paris hospitals. The object of these is to preserve the life of those feeble infants who would die under ordinary circumstances. The machine is in two compartments—one filled with warm water, the other containing a basket lined with wadding, in which the baby is deposited. In the lid of this compartment is a glass pane, to enable the nurse under whose charge the child is placed to watch the development of the incubation, and report its progress to the doctor. It is said, incredible as it sounds, that infants of only half the average weight of new-born children, and otherwise deficient in vitality, who have been reared to maturity in these incubators, have come out of them strong and healthy, and it seems likely that the machines will be generally adopted.

Her Royal Highness the Princess Christian (Princess Helena) honoured the Royal Hospital for Diseases of the Chest, City Road, with a private visit on Saturday afternoon, the 19th inst. Her Royal Highness was received by the Vice-Chairman of the Hospital, Mr. Sidney Gosnell, and by the Secretary, Mr. John J. Austin, and was conducted through the wards, where she spoke kindly and sympathisingly with the patients, and she also visited the hospital chapel. The Princess expressed her regret that some of the wards were closed for lack of funds, and hoped that they might soon be opened. Before leaving Her Royal Highness graciously desired that one of the wards might in future bear her name, and be called the "Helena Ward." The hospital, therefore, will have the great honour of having four royal wards, viz., "Queen Victoria Ward," "Albert Edward Ward," "Beatrice Ward," and "Helena Ward"—the fifth ward bearing the honoured name of "Shaftesbury Ward." Her Royal Highness was accompanied by Colonel Gordon, Lady Tweeddale, the Hon. Mrs. Jeune, Mr. G. T. Bartley, M.P., Mrs. Bartley, &c.

We recently called attention to the opening which India presents to medical women who possess British qualifications to practice. Lord Kinnaird is endeavouring to interest as many people as possible in the work of the Zenana Bible and Medical Mission, which has charge of the medical missions to the people of India. We regret to see, however, that but little praise is bestowed by this society on the efforts which Lady Dufferin is so wisely making to provide women, throughout India, with the medical aid they at present sorely need. Surely the Zenana Mission, which, we have reason to know, is most valuable and worthy of support, might pursue its proper course without stopping to throw, even by implication, a stone at the excellent movement which the Viceroy and his wife are promoting. India is vast enough to leave abundant openings for both of these movements without any risk of overlapping. We would, therefore, counsel Lord Kinnaird to omit his reference to Lady Dufferin's fund as one which does not commend itself to the entire sympathy of those specially interested in Christian missions. We have always supported warmly the Zenana Missions, but in face of recent statements in the *Times* and elsewhere it would strengthen Lord Kinnaird's appeal were he to add to it some statement of the receipts and expenditure, and to show the relation which the latter bears to results in the case of this society.

THE following vacancies are announced this week, the amount of salary in each case being given after the name of the institution :—

**Resident Medical Officers.**

Paddington Green Children's Hospital. House Surgeon.—Salary, £80 per annum; with rooms.
Burton-on-Trent Infirmary. House Surgeon.—Salary, £130; with rooms, coal, and gas.
Bolton Infirmary and Dispensary. Junior House Surgeon.—Salary. £120; with board, &c.
Royal National Hospital for Consumption, Ventnor. Resident Medical Officer.—Salary, £100 per annum; with board, &c.
Stockport Infirmary. Assistant Medical Officer.—Salary, £70 per annum; with board.
Wallasey Dispensary, Liscard, Birkenhead. House Surgeon.—£120 per annum; with furnished residence.
Miller Hospital and Royal Kent Dispensary, Greenwich Road, S.E. Junior Resident Medical Officer.—Salary, £30 per annum; with board.

**Non-resident Medical Officers.**

Great Northern Central Hospital, Caledonian Road, N. Obstetric Physician to Out-patients
Home and Infirmary for Sick Children, Lower Sydenham, S.E. Second Honorary Assistant Physician.
St. George's Hospital. Physician.
St. George's Hospital. Surgeon.
Hull Royal Infirmary. Honorary Dental Surgeon.

**Matron.**

Clifton College Sanatorium.

**Nurses.**

The Denbighshire Infirmary. Wanted a Head Nurse, well trained in Surgical and Medical Work.—Salary to commence at £25.
North Devon Infirmary, Barnstaple. Wanted Certificated Charge Nurse.—Salary, £25.
Bristol Royal Infirmary. Two Nurses (of at least one year's experience).—Wages £16 first year, £18 second.
West Bromwich District Hospital. Surgical and Medical Night Nurse.—£25; uniform.
Blackburn and East Lancashire Infirmary Assistant Nurse.
Gravesend Hospital, Gravesend. Surgical and Medical Night Nurse.—£20; uniform and washing.

**Probationer.**

Blackburn and East Lancashire Infirmary. Probationer.

---

THE Dental Hospital of London, Leicester Square, has received £1,000 from its medical staff and lecturers towards the £5,000 required for the extension of the Hospital, rendered absolutely necessary in consequence of the large increase in the number of patients.

---

IN our advertising columns will be found an account of the "Electrified Rooms" at Southampton. These rooms have been visited and personally inspected, and a notice of them has already appeared in THE HOSPITAL. The rooms mark a new departure in electro-therapeutics, and are well worthy of the attention of the profession; and especially of those who have cases of chronic rheumatism, neuralgia, and allied affections, which are intractable.

---

DURING the past month of October the nursing profession lost two of its members. Miss Storey, Head Sister of the Female Nursing Staff of the Royal Naval Hospital, Gosport, died on the 8th, after a very long and painful illness. She was trained at the Middlesex Hospital, and in October, 1884, was appointed Head Sister of the Royal Naval Hospital, Portsmouth; thence transferred in May of the following year to Gosport. Miss Storey, though suffering severely, continued to discharge her duties until July last, when she was compelled to seek a change. She returned to duty after a two months' visit to Scotland, which, however, failed to restore her health, and after lingering for two weeks at the hospital died as above stated. Miss Storey was greatly respected and loved by those who knew her intimately, as was evidenced not only by her superiors and fellow nurses, but by the presence of the officers and sick berth staff at the funeral at Haslar Cemetery on 11th ult. Miss Lloyd, who was recently matron of the Sidmouth Cottage Hospital Devonshire, and who has done good work in the nursing world, also died in October.

---

MR. T. B. UDALL presided at the annual meeting of the Governors of the North Staffordshire Infirmary, held in the Board-room, Harts' Hill, on Thursday. Dr. Arlidge, Dr. Folker, and Dr. West, with Mr. Kitchener, Mr. Malkin, Mr. Minton, Mr. Lenhouse, Mr. W. A. Adderley, Mr. C. Linum, and Dr. Bullock were also present. The Chairman in moving the adoption of the report, expressed his satisfaction at the fact that this year the institution had been able to pay its way, and that there had been an excess of income over expenditure of £30.

---

CONCERNING male nurses the *City Press* says: "Several correspondents have called attention to the desirability of training male nurses for men. No doubt there are cases in which the strength of a man is required to control the violence of a patient, and others in which the nature of the complaint must be objectionable to women, but I very much doubt whether men would ever be able to do the work half so well. Men have not, as a rule, the tact, patience, and endurance which are essential qualities for a good nurse."

---

THE terrible calamity which occurred recently in connexion with the Exeter Theatre has evidently produced a deep impression upon the inhabitants of that city. A special General Court of the Governors of the Hospital was held a few days ago to consider the question of the means of removing patients from the hospital in case of fire, and also to take steps for the prevention or prompt extinction of fire in case of its occurrence. Hospital authorities in other towns and cities would do well to take into consideration the condition of the institution over which they preside in this respect.

---

THE ninety-fifth annual meeting of the Board of Management of the Belfast Royal Hospital was held on Monday, the Mayor (Sir J. H. Hazlett) presiding. Among those present were: The Venerable Archdeacon Scaevan, the Very Rev. J. P. Green, D.D., the Rev. J. Fordyce, the Rev. J. Costello, the Rev. Dr. Hamilton, Mr. George Fisher, Mr. John Campbell, Mr. R. W. Murray (honorary secretary), and many others. The report stated that during the past year there had been an increase both in general subscriptions and from Church collections, and that upon the whole the financial condition of the hospital was satisfactory. The year commenced with a balance of £1,048 8*s*. 2*d*. on the wrong side, but at the end of twelve months the deficiency is found to be reduced by almost one half. A little increase in the strenuousness of the exertions put forth by the town and neighbourhood will render the hospital entirely self-supporting.

---

AT the Town Hall, Croydon, on Friday evening Sir Thomas Edridge presided at the annual meeting of the Governors of the Hospital. There were also present Dr. Carpenter, J.P., Dr. Adams, Dr. Strong, the Rev. J. M. Braithwaite, Alderman Thrift, Councillor Schmitz, and several other gentlemen. A slight increase of ordinary subscriptions was announced for the year, although the amount received is still insufficient for current expenses. In so far as the results of treatment are concerned the report was eminently satisfactory. We are glad to see that an ophthalmic department has been instituted, and placed under the care of Mr. Charles Wray, F.R.C.S., who will attend every Wednesday morning. The number of dental cases under treatment during the year was no less than 539. It is satisfactory to note that, in spite of the rather small amount of annual subscriptions, the year closed with a balance in the hands of the treasurer of £101 2*s*. 2*d*.

The Committee of the South Ayrshire Needlework Society have this year, according to their usual custom, sent a large parcel of clothing to the Ayr County Hospital.

Renewed complaints reach us of the difficulty of obtaining The Hospital. One lady informs us that she asked her bookseller, in Hackney, for it, and was told that it was no longer published. The news surprised her; it surprises us too. In face of this information as to our decease we venture to state that The Hospital is not only still published, but is sold at no fewer than 600 places in London alone, including all Messrs. Smith's bookstalls; and that we shall be obliged by any subscribers who cannot obtain it communicating with us.

Mr. A. H. Haggard has, we understand, resigned the appointment of Secretary of the London Hospital. This announcement will be received with regret by all who have had business relations with the Secretary of the "London," because they cannot fail to have been struck with his invariable courtesy, nor can they have failed to notice his willingness to aid every good work on all occasions. We hear that Mr. Haggard has decided to devote himself to work at the Bar, and we, in common with the many friends he has made in the hospital world, wish him all prosperity and happiness in his new career.

The vacancy at the London Hospital will not be easy to fill. It affords a great opportunity for a new departure in the method of raising income for this institution. We have always felt that if the artisan and labouring classes, who chiefly supply patients to the wards of the London Hospital, were approached systematically, on the plan adopted by the managers of the Northern and Scotch hospitals, they would cheerfully and largely contribute to the income of that hospital. The first, and probably the most important, consideration is to find the right man to fill Mr. Haggard's place. Everybody who has any feeling of manliness in him must be proud to be connected in any way with the great hospital of East London. Besides, the management and the managers of this institution deservedly possess the confidence of all who know what good management means and can accomplish, and it is an honour, therefore, to be associated with them.

We recently had occasion to compliment the people of Hastings upon the possession of probably the most complete building yet erected for hospital purposes. It was said by Mr. Collins, F.R.I.B.A., in a recent discussion on Hospital Construction, that however exeellent the design and work of an architect might be, it was within the power of the managers to neutralise everything he had attempted in the way of producing a perfect hospital. If what we hear is correct, the Hastings authorities are making the fatal mistake of allowing the views of the matron, many of which have been exploded by experience long since, to upset the plan of the building, by turning the convalescents' room into a children's ward; by retaining possession for her own use of the room which ought to be the children's ward; by converting the single-bed wards into bed-rooms for the head nurses, and generally by similar retrogressive and regrettable proceedings. English visitors to the Antwerp Circular Hospital, whilst regretting the state to which bad administration has reduced the wards in that building, have always reflected that such things were not possible in England. If the people of Hastings decide to allow the management to destroy the efficiency and completeness of their hospital, foreign visitors, who are likely to come in great numbers, will not only have their revenge, but the credit of the English system of hospital administration must suffer also. Might we suggest that some special arrangement should be made to allow experts to see the whole of the hospital when they apply to be permitted to do so? The other day one of the most eminent architects in the profession went to Hastings to make a study of this hospital, and was not afforded reasonable facilities for the purpose, nor was he allowed to see the whole of the buildings.

